# Migraine pathogenesis and state of pharmacological treatment options

**DOI:** 10.1186/1741-7015-7-71

**Published:** 2009-11-16

**Authors:** Till Sprenger, Peter J Goadsby

**Affiliations:** 1 UCSF Headache Group-Department of Neurology, University of California, San Francisco, CA, USA

## Abstract

Migraine is a largely inherited disorder of the brain characterized by a complex, but stereotypical, dysfunction of sensory processing. Often the most obvious clinical symptom is head pain, but non-headache symptoms such as photophobia, phonophobia and nausea are clearly part of the typical presentation. This review discusses the current pathophysiological concepts of migraine and migraine aura, such as a possible brainstem dysfunction and cortical spreading depression. Acute and preventive migraine treatment approaches are briefly covered with a focus on shortcomings of the currently available treatment options. A number of different receptors, such as calcitonin gene-related peptide (CGRP), TRPV1 and glutamate receptors, are currently being targeted by potential novel migraine therapeutics. The prospects of this research are exciting and are likely to improve patient care.

## Introduction

Migraine is a disorder characterized by a broad sensory processing dysfunction. Thereby, the perception of normal sensory stimuli, somatosensory, visual, auditory and olfactory, is thought to be centrally facilitated. This dysfunction has a strong genetic background [1] and leads to the perception of a combination of headache, photophobia, phonophobia, osmophobia and nausea. The disorder was considered to be vascular in origin for much of the 20th century [2], although it was considered to be a disorder of the nervous system by 19th century luminaries [3]. This was related to the pounding or throbbing nature of the pain and triggering by vasoactive substances that now seem more likely to be due to the prominent perception of pain in the context of the dense somatosensory innervation of intracranial vessels. This view is supported by data which include the fact that: vasoactive intestinal peptide - a strong intracranial vasodilator - does not trigger migraine [4]; intracranial vasodilatation also occurs secondary to experimental head pain stimulation [5], probably mediated by the trigeminal-parasympathetic reflex; and non-vasoconstrictor drugs, such as aspirin [6] and calcitonin gene-related peptide (CGRP) receptor antagonists can abort migraine attacks [7]. Most importantly, headache is only one of the neurological symptoms of migraine, where the dysfunction can really only be located in the brain itself.

This article provides an update on potential mechanisms of migraine and aura pathogenesis and reviews current and future medical strategies for the acute and preventive treatment of migraine.

## Aura and cortical spreading depression

A subgroup of migraineurs experience aura, typically before the onset of head pain, with some of their attacks and there are several lines of evidence that cortical spreading depression is the pathophysiological substrate. In early clinical observations, it was noted that the progression of aura symptoms is consistent with a process transiently compromising cortical function at a speed of about 3 mm per minute [8]. Leao suggested that cortical spreading depression (CSD), advancing at the identical speed over the cortex, was the electrophysiological correlate of visual aura in humans. By now, the existence of CSD in humans has been proven using electrophysiological methods [9-11] and human imaging studies [12,13]. A possible link between CSD and headache has been provided by the observation that CSD can activate trigeminal meningeal afferents [14], although contradictory data also exist [15]. Hence, CSD could not only induce aura symptoms, but also explain the head pain in patients with aura. This view is not supported by recent controlled trials which show that tonabersat, a possible gap-junction blocker and inhibitor of CSD [16], does not prevent migraine headache [17] but can prevent migraine aura [18]. It has been suggested that CSD also has a role in migraine without aura but the tonabersat studies suggest this is less likely. Silent aura, the occurrence of CSD confined to regions not clinically eloquent and still activating trigeminal afferents, is a tempting concept when looking for a unifying concept of migraine with and without aura. However, as noted above in the study by Hauge *et al*. [18], tonabersat was ineffective in migraine without aura and, as the drug reduced the frequency of aura attacks, this result clearly challenges the concept of the silent aura [18]. Moreover, in a recent case series, three patients were described who reported that their auras resolved when migraine preventives were started, while in parallel they experienced a worsening of the frequency of their migrainous headaches [19]. Finally, the sequence of a migraine attack has probably already been initiated long before the actual onset of CSD and aura. Migraine attacks often start with a typical premonitory phase when patients complain of tiredness, reduced concentration, irritability, yawning and other non-headache symptoms hours to days before the onset of aura and headache [20,21]. At this point, many patients can predict the onset of a full-blown migraine attack and the start with non-headache symptoms underlines that migraine is much more than an isolated pain disorder.

## Neurogenic plasma protein extravasation

It has been suggested that some component of the migrainous pain is related to dural plasma protein extravasation with sterile neurogenic inflammation [22]. Electrical stimulation of the trigeminal ganglion induces plasma protein extravasation and this can be blocked by some of the substances which are active in acute migraine attacks (for example, sumatriptan) [23]. On the other hand, a blockade of neurogenic plasma protein extravasation is not completely predictive of anti-migraine efficacy, as other compounds which are effective in this experimental model of migraine, such as neurokinin-1 receptor antagonists, have failed in clinical migraine prevention trials [24,25]. Hence, plasma protein extravasation seems to be an epiphenomenon rather than a pivotal mechanism of trigeminal activation and migraine generation [26].

## Brainstem dysfunction in migraine pathogenesis?

In our view, the whole clinical picture of migraine can be better explained by a dysfunction of neuromodulatory structures in the brainstem, such as the locus coeruleus or periaqueductal grey matter. The locus coeruleus, the major noradrenergic nucleus, has a critical role in the regulation of cortical function and is known to modulate responses to afferent traffic [27]. Its connections are widely distributed throughout the neocortex and in positron emission tomography (PET) studies investigating acute migraine attacks, activation of an area of the dorsolateral brainstem that included the locus coeruleus has been shown [28,29], although there is some remaining uncertainty regarding the exact anatomical location of this activation because of the limited spatial resolution of PET. Compared to other hypothesis on migraine neurobiology, dysfunction of such brainstem structures and networks could not only account for the somatosensory component of migraine (headache) but also for the auditory, olfactory and visual components. Moreover, a locus coeruleus dysfunction could also explain the distractibility and anxiety [27] which is often observed in migraineurs.

## Pharmacological and interventional strategies of migraine prevention

Migraine prevention is an important component of therapy aimed at reducing the attack frequency and severity. Unfortunately, the mechanisms of action of current preventives are not well understood. A potential mechanism is the inhibition of cortical spreading depression but, as noted above, the efficacy against cortical spreading depression does not necessarily predict the efficacy in treating migraine without aura [18]. Substances that have proven beneficial in migraine, with and without aura, broadly comprise compounds from the following classes: beta-blockers (for example, propranolol), antidepressants (for example, amitriptyline), anticonvulsants (for example, valproate, topiramate), calcium channel blockers (for example, flunarizine) and serotonin antagonists (for example, methysergide) [30]. According to the pathophysiological concepts discussed above, these drugs most probably target the activity of modulatory circuits as well as the neuronal activity in afferent sensory pathways such as the trigeminal system [31]. Although many patients can be effectively managed using the available substances, side effects and contraindications because of co-morbidities can complicate treatment. A particular problem is the prediction of which patients will respond to which substance as treatment is often largely conducted by trial and error, which is frustrating for the patients and treating physicians alike. In order to obtain a better understanding of the mechanisms of the action of the available substances, phenotype-driven treatment approaches [32] as well as pharmacogenomic considerations [33], might help to overcome such issues. Moreover, newer treatment strategies, such as the use of Botulinum toxin in migraine prevention, are currently undergoing clinical trials (Table 1). Interventional neuromodulational approaches are also promising, including the stimulation of the occipital nerve, where functional imaging studies show that the central processing of pain signals in migraine in the thalamus may be modified by such therapies [34]. Studies, such as ONSTIM [35] and PRISM [36], are now complete with their implications being analysed (PRISM NCT00286078 and [37-39]). This exciting and developing area might be especially helpful in the treatment of medically refractory patients.

**Table 1:** Selected migraine drug development programs (compiled from http://www.clinicaltrials.gov).

Drug	Mechanism of action	Stage of development
**Acute attack**		

Telcagepant (MK-0974)	CGRP receptor antagonist	Phase III- complete

BI 44370	CGRP receptor antagonist	Phase II

COL-144	5-HT_1F _receptor agonists	Phase II

SB-705498	TRPV1 receptor antagonists	Phase II

BGG492	AMPA receptor antagonist	Phase II

Tezampanel (LY-293558)	AMPA and kainate receptor antagonist	Phase II

LY466195	GLUK5 kainate receptor antagonists	Phase II

NPS 1776	Anticonvulsant	Phase II

BGC20-1531	Prostanoid EP4 receptor antagonist	Phase II

NXN-188	nNOS inhibition and 5-HT_1B/D _agonist	Phase II

**Preventives**		

Ramelteon	Melatonin MT1 and MT2 receptor agonist	Phase IV

Botulinum Toxin type A	Unknown	Phase III

Tonabersat	Gap junction/CSD inhibitor	Phase II- failed in migraine without aura

ADX10059	mGluR5 Modulator	Phase II

Perampanel (E2007)	AMPA receptor antagonist	Phase II

GW274150	iNOS	Phase II- failed

Carisbamate (RWJ-333369)	Anticonvulsant	Phase II- failed

CGRP, calcitonin gene-related peptide; CSD, cortical spreading depression; nNOS, nitric oxide synthase; iNOS, inducible nitric oxide synthase

## Pharmacological strategies for migraine attack treatment

Treatments for acute attacks can be divided into non-specific anti-pain compounds, such as simple analgesics and NSAIDS, and more migraine-specific treatment approaches, such as ergot-derivates and triptans, which are active at 5-HT_1 _receptors. Stratified care, the choice of an acute medication on the basis of attack characteristics, has been shown to be superior to stepped care [40] and, as triptans are most effective, a triptan is typically chosen for the more severe migraine attacks. Triptans, because of their better tolerability, have replaced ergotamine in most cases [41]. Sumatriptan and its six licensed successors are agonists at 5HT_1B/D _receptors. They reduce neuronal activity via these receptors at the trigeminocervical complex [42] and thalamic level [43] and these areas, instead of the blood vessels, are most probably also important sites for their therapeutic action in migraine. There are still situations where tolerability and contraindications to use are a problem. The main issue for triptans relates to their vasoconstrictor properties and related cardiovascular and cerebrovascular safety concerns. This necessitates that triptans are not used in patients with cerebrovascular or cardiovascular contraindications [44]. The new group of so-called *Gepants*, CGRP receptor antagonists, may soon offer an option to such patients. Two substances of this class, parenteral olcegepant [7] and oral telcagepant [45], have proved to be effective in the acute treatment of migraine without vascular liability [46]. Their effectiveness seems to be comparable to triptans [45].

The announcement of the success of a phase II dose-ranging proof-of-concept study with the 5-HT_1F _receptor agonist CO-144 offers another prospect for a non-vasoconstrictor acute anti-migraine therapy [47]. The success of CGRP receptor antagonists and 5-HT_1F _receptor agonists reinforce a neurally-based approach to migraine and emphasize that migraine is a brain disorder. Other strategies, such as nitric oxide synthase inhibitors [48], vanilloid TRPV1 receptor antagonists [NCT00269022, [49]] and glutamate, AMPA/kainate receptor [50] as well as pure kainate receptor antagonists, will hopefully follow providing an even broader repertoire of migraine-specific drugs (Table 1, [16,51]).

## Conclusion

Migraine is a disorder of the brain characterized by a complex sensory dysfunction. Hence, therapeutic approaches with neural targets are most promising. Currently, a number of such pharmacological and interventional therapies are being investigated and it is likely that patients can benefit from CGRP receptor antagonists and occipital nerve stimulation in the near future. In addition to these new treatment strategies, defining patient populations who are more likely to respond to the already available therapeutic options also has great potential to improve patient care in daily practice.

## Abbreviations

CGRP: calcitonin gene-related peptide; CSD: cortical spreading depression; PET: position emission tomography.

## Competing interests

TS declares no competing interests. PJG has consulted for, advised or collaborated with Advanced Bionics, Allergan, Almirall, ATI, AstraZeneca, Belgian Research Council, Boehringer-Ingelheim, BMS, Boston Scientific, Colucid, Eli-Lilly, Fidelity Foundation, GlaxoSmithKline, Johnson & Johnson, Kalypsys, Medtronic, MAP, Migraine Research Foundation, Migraine Trust, Minster, Medical Research Council-UK, MSD, NINDS, Netherlands Research Council, Neuralieve, Neuraxon, NTP, Organisation for Understanding Cluster Headache-UK and Pfizer.

## Authors' contributions

TS and PJG each participated in the writing and editing of this minireview. Both authors have read and approved the final manuscript.

## Pre-publication history

The pre-publication history for this paper can be accessed here:

http://www.biomedcentral.com/1741-7015/7/71/prepub
